# An Inter-Continental American Medical Congress

**Published:** 1891-06

**Authors:** 


### Society Notes.

### The Inter-Continental American Medical Congress.

OFFICE OF THE PERMANENT SECRETARY
OF THE AMERICAN MEDICAL ASSOCIATION,
*Philadelphia, June 4th, 1891.*

*To the Medical Profession of The Western Hemisphere:*

At a meeting of the American Medical Association, held at Washington, May 5th, 1791, Dr. Charles A. L. Reed, of Cincinnati, introduced the following:

*Resolved*, That the American Medical Association hereby extends a cordial invitation to the medical profession of the Western Hemisphere, to assemble in the United States in an Inter-Continental American Medical Congress.

*Resolved*, That the Committee on Nominations be and is hereby instructed to nominate one member for each State and Territory, and one each from the army, navy, and marine hospital service, who shall constitute a committee, which is hereby instructed to effect a permanent organization of the proposed Inter-Continental American Medical Congress, and to determine the time and place at which the same shall be held.

The resolutions were seconded by Dr. Wm. H. Pancoast and others,. and unanimously adopted.

Pursuant to the foregoing, a committee composed of the following physicians was nominated and elected :

Alabama, W. H. Sanders; Arizona, Henry A. Hughes; Arkansas, Ed Bentley; California, W. R. Cluness; Colorado, Wm. A. Campbell; Connecticut, C. A. Lindsley; Delaware, C. H. Richards; District of Columbia, D. W. Prentiss; Florida. C. R. Oglesby; Georgia, J. McFadden Gasten; Idaho, Geo. P. Haley; Illinois, N. S. Davis; Indiana, A. M. Owen; Iowa, B. H. Criley; Kansas, J. E. Minney; Kentucky, J. N. McCormack; Louisiana, Stanford E. Chaille; Maine, Hampton E. Hill; Maryland, Geo. H. Rohe; Massachusetts, Augustus P. Clarke; Michigan, C. Henri Leonard; Minnesota, P. H. Millard; Mississippi, W. T. Kendall; Missouri, I. N. Love; Montana, Thos. J. Murray; Nebraska, R. C. Moord; Nevada, P. J. Aiken; New Hampshire, Irving A. Watson; New Jersey, E. J. Marsh; New Mexico, C. E. Winslow; New York, John Cronyn; North Carolina, H. Longstreet Taylor; North Dakota, E. M. Darrow; Ohio, Charles A. Reed; Oregon, Wm. Boys; Pennsylvania, Wm. Pepper; Rhode Island, Geo. L. Collins; South Carolina, R. A. Kinloch; South Dakota, J. W. Freeman; Tennessee, J. R. Buist; Texas, J. W. Carhart; Utah, F. S. Bascom; Vermont, H. H. Holton, Virginia, J. S. Wellford; Washington, J. M. Morgan; West Virginia, J. H. Brownfield; Wisconsin, J. T. Reeve; Wyoming, J. H. Finfrock; United States Navy, A. L. Gihon; United States Marine Hospital Service, J. B. Hamilton.

WM. T. BRIGGS, President.

WILLIAM B. ATKINTON, Permanent Secretary.

THE INTER-CONTINENTAL AMERICAN CONGRESS.
OFFICE OF THE CHAIRMAN
OF THE COMMITTEE ON PERMANENT ORGANIZATION.

*Cincinnati, June 6, 1891.*

The committee appointed by the American Medical Association to effect a permanent organization of the Inter-Continental American Medical Congress, met at "The Arlington," Washington, May 7, 1891. The following officers were elected :

Charles A. L. Reed, M. D., Cincinnati, O., Chairman; J. W. Carhart, M. D., Lampasas, Texas, Secretary; I. N. Love, M. D., St. Louis, Mo., Treasurer.

On motion, the officers were appointed a special committee to draft a constitution and report the same at an adjourned meeting of the general committee, to be held at St. Louis, Mo., Wednesday, October 14, 1891, when the time and place of meeting of the congress will be decided and permanent officers be elected.

CHARLES A. L. REED, M. D., Chairman.

J. W. CARHART, M. D., Secretary.

